# Dietary Supplementation with Rumen-Protected Arginine or N-Carbamylglutamate Enhances Fetal Liver Development in Nutrient-Restricted Pregnant Hu Ewes

**DOI:** 10.3390/ani14131988

**Published:** 2024-07-05

**Authors:** Yuexia Lin, Lingwei Sun, Mengqian He, Jiehuan Xu, Caifeng Wu, Jun Gao, Jianjun Dai

**Affiliations:** 1 Shanghai Municipal Key Laboratory of Agri-Genetics and Breeding, Institute of Animal Husbandry and Veterinary Science, Shanghai Academy of Agricultural Sciences, Shanghai 201106, China; lyxia05@163.com (Y.L.); sunlingwei1987@126.com (L.S.); he1037247863@163.com (M.H.); jiehuanxu810@163.com (J.X.); wucaifengwcf@163.com (C.W.); 2 Key Laboratory of Livestock and Poultry Resources (Pig) Evaluation and Utilization, Ministry of Agriculture and Rural Affairs, Shanghai 201106, China

**Keywords:** maternal undernutrition, rumen-protected arginine (RP-Arg), N-carbamylglutamate (NCG), fetal liver function, antioxidant, apoptosis

## Abstract

**Simple Summary:**

Intrauterine growth restriction (IUGR), defined as the impaired growth and development of a mammalian embryo/fetus or fetal organs during pregnancy, is a significant public health concern worldwide. The fetus is sensitive and responsive to the maternal milieu, and there is evidence that maternal nutritional deficiency has the potential to affect fetal liver development. N-carbamylglutamate (NCG), a stable activator of endogenous Arg synthesis, has been demonstrated to improve fetal survival by increasing the endogenous synthesis of arginine. In this study, we attempted to determine whether supplementation with rumen-protected arginine (RP-Arg) or NCG during late pregnancy in Hu ewes improves fetal liver function by suppressing fetal hepatic abnormalities and apoptosis. We found that maternal undernutrition during pregnancy leads to the maldevelopment of the fetal liver. However, supplementation with RP-Arg and NCG can enhance fetal liver development under such conditions.

**Abstract:**

This study was conducted in nutrient-restricted pregnant Hu ewes to determine whether rumen-protected arginine (RP-Arg) or N-carbamylglutamate (NCG) supplementation affects fetal liver growth and development. From 35 d to 110 d of gestation, 32 Hu ewes were randomly divided into four groups: a control group (100% of the National Research Council (NRC) requirements), a nutrient-restricted group (50% of the NRC requirements), and two treatment groups (ARG and NCG, 50% of the NRC requirements, supplemented with 20 g/day RP-Arg or 5 g/day NCG, respectively). Fetal body weights, fetal liver growth performance, the capability of antioxidation, and the expression of the mRNA and proteins of apoptosis-related genes in the fetal liver were determined and analyzed at 110 d of gestation. The dry matter, water, fat, protein, and ash components of the fetal livers in the RG group were found to be lower than in the CG group, and these components were significantly higher in the NCG group than in the RG group (*p* < 0.05). A decrease in DNA, RNA, and protein concentrations and contents, as well as in protein/DNA ratios, was observed in the RG group in comparison to the CG group (*p* < 0.05). Compared with the RG group, the NCG group had higher concentrations of DNA, RNA, and protein, as well as higher protein/DNA ratios (*p* < 0.05). The RG group had lower concentrations of cholinesterase, nitric oxide, nitric oxide synthase, superoxide dismutase, alanine aminotransferase, and total protein than the CG group (*p* < 0.05). The RG group had higher levels of glutathione peroxidase, maleic dialdehyde, and aspartate aminotransferase than the CG group (*p* < 0.05). In the RG group, the mRNA and protein expression of p53 and Bax was significantly increased (*p* < 0.05) compared with the CG group, and the gene expression of FasL and Bcl-2, the ratio of Bcl-2 to Bax, and the protein expression of Bcl-2 in the RG group were lower (*p* < 0.05) than in the CG group. It appears that RP-Arg and NCG supplementation during pregnancy could influence fetal liver growth and development. A nutrition-based therapeutic intervention to alleviate reduced fetal growth can be developed based on this study, which has demonstrated that maternal undernutrition during pregnancy induces the maldevelopment of the fetal liver.

## 1. Introduction

Maternal undernutrition in pregnant animals is a major concern worldwide, and it is known to cause fetal intrauterine growth retardation (IUGR) [1]. During fetal development, the liver is a major site of various metabolic and secretory functions [2]. Maternal nutritional deficiency and undernutrition have been observed to impair the growth of fetal livers [3]. However, the precise mechanisms responsible for cell growth, hepatic antioxidation, and the anti-apoptosis activities of the fetal liver under maternal feeding restrictions remain unknown.

A previous study has shown that the de novo synthesis of L-arginine in ruminants is often not enough to meet the particular requirements of the mother and fetus, especially during late pregnancy, or for high-production ruminant animals [4,5]. Supplemental rumen-protected arginine (RP-Arg) has been proven to enhance the reproductive performance of ewes [6]. N-carbamylglutamate (NCG) improves litter size and fetal survival via increases in the endogenous synthesis of arginine [7]. Researchers found that RP-Arg or NCG supplementation in pregnant ruminants prevented fetal mortality and maintained internal homeostasis [4]. In addition, the cost of chemically synthesized NCG is significantly lower than that of RP-Arg [8]. Our research group previously carried out some progressive work on topics relating to maternal and placental development [9], the concentrations of metabolites and hormones in maternal serum [10], metabolic profiling (metabolomics) in umbilical venous plasma [11], and fetal growth [12]. In the future, we will also explore more aspects of the effects of IUGR on fetuses as well as lambs after birth. However, the understanding of the effects induced by RP-Arg- and NCG-related pathways in fetal liver development in cases of maternal undernutrition remains limited.

Consequently, we hypothesized that supplementing the feed of Hu ewes with RP-Arg or NCG during late pregnancy could improve the liver function of their offspring by suppressing fetal hepatic abnormalities and apoptosis.

## 2. Materials and Methods

### 2.1. Animals and Experimental Design

Jiangyan Experimental Station in Taizhou, Jiangsu Province, purchased 48 multiparous Hu sheep (40.1 ± 1.2 kg body weight) of the same age (18.5 ± 0.5 months of age). All ewes were provided with clean water and fed a diet based on corn and soybean meal after requisite anthelmintic treatments. The detection of the synchronization estrus of the ewes was followed by artificial insemination using frozen–thawed semen from the same ram (0 d of gestation; 0 dG). At 35 dG, 32 ewes carrying two fetuses were randomly selected after identifying the pregnancy using transabdominal ultrasonography, and these individuals were randomly assigned to four groups (eight individuals for each group).

Based on the nutritional requirements for sheep outlined by the NRC (2007) [13], the ewes were randomly divided into the following groups: a control group (100% of the National Research Council (NRC) requirements), a nutrient-restricted group (50% of the NRC requirements), and 2 treatment (ARG and NCG) groups (50% of the NRC requirements, supplemented with 20 g/day RP-Arg or 5 g/day NCG, respectively). The RP-Arg and NCG were purchased from Feeding Feed Science Technology and the Chinese Academy of Sciences, respectively. The dietary composition of the feed provided to all ewes is listed in Supplementary Table S1, and included wild rye, corn, and soybean meal for forage. In accordance with previous studies [4,14,15], the RP-Arg and NCG doses were calculated based on 50% intestinal availability. Therefore, 10 g/day of RP-Arg and 2.5 g/day of NCG were added, respectively. Water was made freely available to the pregnant ewes, and they were fed once daily at 0800 h.

### 2.2. Sample Collection, Handling, and Chemical Components

All ewes were slaughtered at 110 dG, and the blood samples (5 mL) from the fetal umbilical vein were collected in a sterile tube. The blood samples were centrifuged at 3000 rpm for 15 min at 4 °C to obtain the serum. Until the subsequent analyses, the supernatant was stored at −80 °C. Our group has previously documented the major organ characteristics of the mother and fetus [9,10]. To study the development of the fetal liver, this study only examined fetal body and liver weights. As per a previous description [16], each fetus (16 fetuses/group) had the left lateral lobe of its liver snap-frozen in liquid nitrogen, and then stored at −80 °C until later analysis.

The samples were primarily freeze-dried to a constant weight (Christ, Alpha 1-4 lsc, Osterode am Harz, Germany), and then each liver sample was subsequently analyzed for dry matter (DM), crude protein, crude fat, and crude ash contents according to AOAC (2003) [17].

### 2.3. Liver Cellularity Estimates

Freshly thawed fetal liver samples (approximately 1 g) were homogenized and analyzed for DNA, RNA, and protein concentrations. Diphenylamine [18] and Orcinol [19] procedures were used to analyze the DNA and RNA. As a standard curve, bovine serum albumin (Fraction V, Sigma Chemical, St. Louis, MA, USA) was used to determine the total protein concentrations. The DNA concentration was used as an index of hepatic hyperplasia, while ratios of RNA/DNA and protein/DNA were used as an index of liver hypertrophy and potential cellular activity, respectively [16]. The calculation of DNA, RNA, and protein concentrations in the livers was carried out by multiplying the concentrations of DNA, RNA, and protein by the total weight of the liver [20].

### 2.4. Detection of Fetal Liver Functional Indicators and Antioxidation Ability

The measurement of liver enzymes in the blood is routinely used to assess liver function [21]. Analyses of fetal serum alanine aminotransferase (ALT), aspartate aminotransferase (AST), and total protein (TP) were carried out using an automated analyzer (Beckman Coulter Inc., Fullerton, CA, USA) at 37 °C. This analyzer determined the liver enzyme activities of AST and ALT via enzymatic–kinetic methods. Serum TP concentration was determined using the biuret method. The liver samples were homogenized in 5 mL of 0.85% chilled normal saline, centrifuged at 3000 rpm for 10 min at 4 °C, and the supernatants stored at −80 °C for biochemical tests. Subsequent measurements (cholinesterase (CHE), glutathione peroxidase (GSH-Px), activities of monoamine oxidase (MAO), maleic dialdehyde (MDA), nitric oxide (NO), nitric oxide synthase (NOS), superoxide dismutase (SOD) and total antioxidant capacity (TAC)) were determined using commercial kits (Diagnostic Product, Nanjing Jiancheng Bioengineering Institute, Nanjing, China). In all cases, these kits were utilized according to the manufacturer’s instructions. Analyses were conducted within a single assay; the CVs for duplicate samples were all less than 10%, and they were less than 15% between assays.

### 2.5. Sample Preparation for Histology Staining

A series of adjacent 5 µm thick, paraffin-embedded sections of fetal liver samples were cut for the histochemical studies. Using an ApopTag Plus Peroxidase In Situ Apoptosis Detection Kit (Chemicon, Billerica, MA, USA), TUNEL staining was performed to detect apoptosis in liver cells. To estimate the number of total cells and apoptosis cells, two representatives per section were selected and overlaid with a 10 × 10 grid in ImageJ (Bethesda, MD, USA). In the fetal livers, the apoptosis percentage was expressed as the average number of apoptotic cells divided by the average number of total cells [22].

### 2.6. Quantification of Hepatocellular-Apoptosis-Regulating Factors

Quantitative real-time reverse transcription polymerase chain reaction (qRT-PCR) was used to identify several hepatocellular-apoptosis-regulating factors. Here, we chose some hepatocellular-apoptosis-regulating factor genes to measure the following indications: protein 53 (p53), factor-associated suicide (Fas), fas ligand (FasL), B cell lymphoma 2 (Bcl-2), and Bcl-2-associated X protein (Bax). We extracted total cytoplasmic RNA from the fetal liver tissue using TRIzol (Invitrogen, Carlsbad, CA, USA) as directed by the manufacturer. The RNA quality of each sample was determined using spectrophotometric analysis (OD 260/280) and quantified at 260 nm. Using a Revert Aid First-Strand cDNA Synthesis Kit, cDNA was synthesized from the total RNA (2 µg). A summary of the primer sequences used for qRT-PCR analysis can be found in Table S2. The assays were performed using SYBR Green PCR master mix (Applied Biosystems, Foster City, CA, USA) in a total volume of 20 µL on a 7900HT Fast Real-Time PCR System (Applied Biosystems, Foster City, CA, USA). A total volume of 20 µL of PCR mixture was used, along with 10 pM forward and reverse primers, as well as 1.0 µL template cDNA. In this experiment, the following cycling conditions were employed: initial denaturation at 95 °C for 10 min, 40 cycles at 95 °C for 15 s, 10 s at the annealing temperature (Table S2), and 30 s of extension at 72 °C. An amplification curve was generated via dissociation to verify the specificity. The gluceraldehyde-3-phosphate dehydrogenase (GAPDH) gene was regarded as a housekeeping gene, thereby obtaining the threshold cycle for each gene for each sample individually. The intensity ratios between GAPDH and the target genes were used to calculate the relative gene expressions.

### 2.7. Quantification of Apoptosis-Related Regulating Proteins

The homogenized fetal liver tissues were incubated in RIPA lysis buffer (20 mM Tris-HCl, 150 mM NaCl, 1% NP-40, 0.01% Tween-20) for 20 min. A BCA protein assay kit was used to determine the protein concentration in homogenates centrifuged for 15 min at 4 °C. Using electroblotting, approximately 40 μg of proteins were separated via 10% sodium dodecyl sulfate–polyacrylamide gel electrophoresis (SDS-PAGE) under reducing conditions. After blocking with 5% of nonfat dried skim milk for 1 h at room temperature, the membranes were incubated with different primary antibodies (anti-p53, 1:1000; anti-Bcl-2, 1:1000; and anti-Bax, 1:800) overnight at 4 °C. Tris-buffered saline–Tween (TBST) was used to rinse the membranes three times every five minutes, and an anti-rabbit secondary antibody was incubated for 1 h at room temperature after being rinsed three times. ECL Plus reagents (Amersham Biosciences, New York, NY, USA) were used by Amersham Biosciences to detect the immunoreactive protein band after three washes with TBST. An enhanced chemiluminescence detection system (Fujifilm, Tokyo, Japan) and BandScan 5.0 image analysis software (Glyko Inc., Novato, CA, USA) were used to measure band intensities on the membranes. As an internal normalization, β-actin was used to calculate the relative expression; therefore, it was discarded from our experimental design. The statistical model only considered the effect of different diets provided to the pregnant ewes. The Mann–Whitney test was used to analyze data that were not normally distributed. Multiple comparisons between different groups were performed using one-way ANOVA, with the Bonferroni correction applied to the adjustment. The final results were presented as means and standard error of the mean (SEM), with *p*-values < 0.05 indicating statistical significance.

### 2.8. Statistical Analysis

Statistical analysis was performed using the SPSS 17.0 software (SPSS, Inc., Chicago, IL, USA). The amount of variation in fetal sex in this study was found to be non-significant; therefore, it was discarded from our experimental design. The statistical model only considered the effects of different diets of pregnant ewes. Data that were not normally distributed were analyzed using an equivalent nonparametric test (Mann–Whitney test). Multiple comparisons among different groups were explored using one-way ANOVA, and Bonferroni correction was used for any adjustments. Finally, the results were presented as means ± standard error of the mean (SEM), and a *p*-value < 0.05 indicated statistical significance.

## 3. Results

### 3.1. Basic Chemical Components of Fetal Liver

Compared to the RG group, the fetal body weights in the RG group were significantly lower than those in the CG group (*p* < 0.05), while increases were observed in the ARG and NCG groups (*p* < 0.05; Table 1). The fetal liver weights in the RG groups were significantly reduced (*p* < 0.05) compared to those in the CG group, and increased liver weights were observed in the NCG group compared to the RG group. Additionally, both the RG and ARG groups had similar liver weights (*p* > 0.05). The ratio of fetal liver weight to fetal body weight was significantly increased (*p* < 0.05) in the RG group compared to the CG, ARG, and NCG groups, but no differences (*p* > 0.05) were observed among the CG, ARG, and NCG groups.

The DM, water, fat, protein, and ash components of the fetal livers in the RG group were found to be lower than those in the CG group. The DM, water, fat, protein, and ash components of the fetal livers were significantly higher (*p* < 0.05) in the NCG group than in the RG group, but there were no differences between the ARG and RG groups except for the ash component. A lower ash component was observed in the RG group, compared to the other groups. In contrast, the fetuses in the RG, ARG, and NCG groups had greater percentages (*p* < 0.05) of DM, fat, and protein than those in the CG group, and the RG, ARG, and NCG groups exhibited the same trends for DM and protein in the fetal livers (*p* > 0.05). In the fetal livers in the RG, ARG, and NCG groups, there were no differences (*p* > 0.05) in terms of DM or protein content. The percentage of protein was significantly higher (*p* < 0.05) in the ARG group, compared to the CG and NCG groups. However, no differences (*p* > 0.05) were found in terms of the percentage of water or ash among the groups.

### 3.2. Fetal Liver Cellular Indicators

The concentrations, contents, and ratios of the DNA, RNA, and protein from the fetal livers are summarized in Table 2. In the RG group, the DNA, RNA, and protein concentrations and contents, as well as the protein/DNA ratios, were lower (*p* < 0.05) than in the CG group. There were no differences between values in the RG and ARG groups, except for the DNA and protein concentrations. Additionally, no differences (*p* > 0.05) were observed in the fetal liver DNA and RNA contents or the RNA/DNA among the four groups.

### 3.3. Antioxidation Activities of the Fetal Liver and Liver Function Indicators

As shown in Table 3, the RG group had lower levels of CHE, NO, NOS, TAC, and SOD in its liver than the CG group (*p* < 0.05), and the RG group also had higher levels of GSH-Px and MDA (*p* < 0.05). In contrast, the ARG and NCG groups had higher liver concentrations of CHE, NO, and TAC, while GSH-Px and MDA were lower than in the RG group (*p* < 0.05). No significant difference was observed between the groups in terms of the level of MAO in the liver (*p* > 0.05).

The fetal serum TP, AST, and ALT are presented in Table 3. It was found that the RG group’s fetal serum concentration levels for ALT and TP were lower than those in the CG group (*p* < 0.05), whereas the RG group’s fetal serum concentration of AST was higher (*p* < 0.05). The NCG and ARG groups both showed higher ALT concentrations than the RG group (*p* < 0.05), but the AST concentrations were lower. Although the ALT concentrations in both the CG and NCG groups were higher than those in the ARG group (*p* < 0.05), the AST concentrations were lower (*p* < 0.05) in both groups, compared to those in the ARG group.

### 3.4. TUNEL Staining to Identify Apoptotic Cells

**Figure 1:**
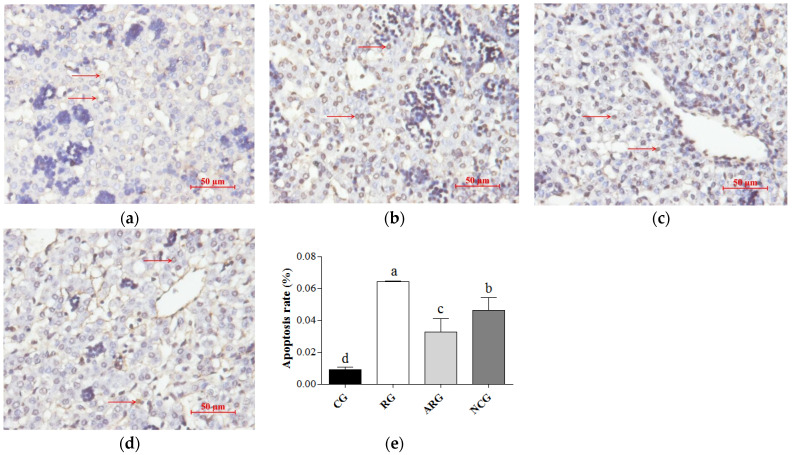
Apoptosis of fetal liver cells. (**a**–**d**) TUNEL staining was used to detect apoptosis in liver cells (red arrow) from CG, RG, ARG, and NCG groups, respectively. (**e**) Apoptosis of fetal liver cells. Bars = 50 µm. Different letters above bars indicate significant differences (*p* < 0.05).

A TUNEL assay highlighted the apoptotic cell nuclei of the liver cells; the percentages of apoptotic cells are presented in Figure 1. The fetuses in the RG group had the highest apoptotic cell percentage among the groups. A significantly lower percentage of apoptotic cells was detected in the ARG and NCG groups, compared to the RG group (*p* < 0.05), but there was a greater percentage of apoptotic cells in the ARG and NCG groups relative to the CG group (*p* < 0.05). However, we also found a higher percentage of apoptotic cells in the fetal livers from the ARG group than in the NCG group.

### 3.5. Apoptosis Genes and Protein Expression

**Figure 2:**
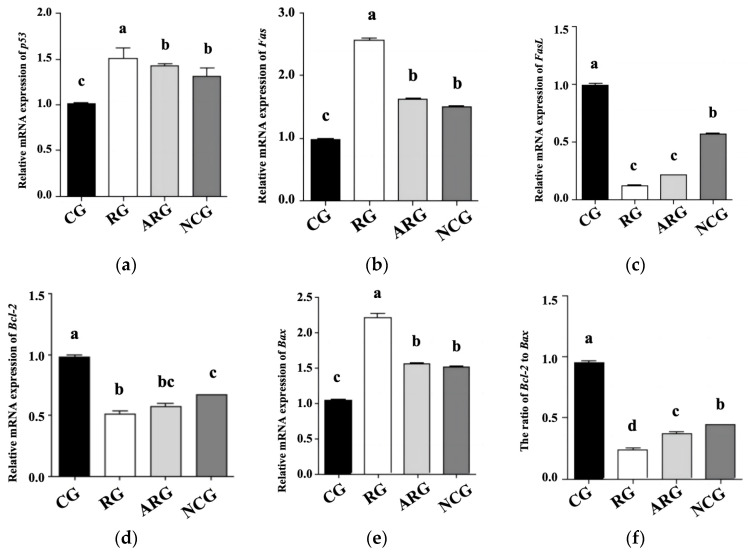
mRNA expression of genes associated with hepatic apoptosis of fetal liver. (**a**) p53, (**b**) Fas, (**c**) FasL, (**d**) Bcl-2, (**e**) Bax (**f**) Bcl2 to Bax. Different letters above bars indicate significant differences (*p* < 0.05).

Figure 2 shows the qRT-PCR analysis of *p53*, *Fas*, *FasL*, *Bcl-2*, and *Bax* mRNA levels to determine their role in fetal hepatocellular apoptosis regulation. Compared with the CG group, the RG group’s mRNA expression levels of *p53*, *Fas*, and *Bax* were significantly higher (*p* < 0.05), while those of the ARG and NCG groups were significantly lower (*p* < 0.05). In comparison with the controls, the *FasL* and *Bcl-2* gene expression levels were significantly lower (*p* < 0.05) in the RG group. Compared with NCG group, the *FasL* and *Bcl-2* mRNA expression and the *Bcl-2* to *Bax* ratio in the RG group were decreased (*p* < 0.05), while *FasL* and *Bcl-2* mRNA expression showed no effect (*p* > 0.05) in the RG group compared with the ARG group. The liver mRNA expression levels of *p53*, *Fas*, *Bcl-2*, and *Bax* were not significantly different between the ARG and NCG groups (*p* > 0.05), except for *FasL* mRNA expression and the Bcl-2 ratio.

**Figure 3:**
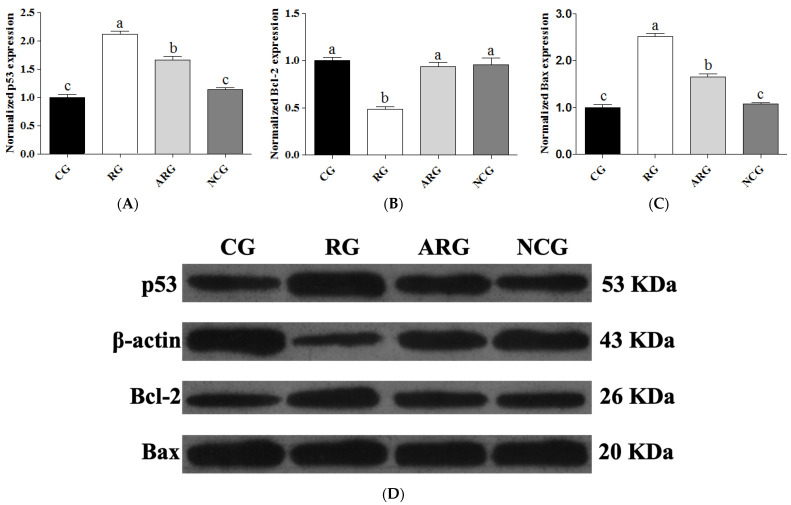
Relative expression of p53 (**A**), Bcl-2 (**B**), and Bax (**C**) protein. (**D**) The cropped images from Western blot analysis. Anti-β-Actin antibody was used to assure equal target protein amounts between the samples. Each bar denotes mean ± SEM. For the same protein, different letters (a–c) indicate *p* < 0.05.

Western blot analysis was used to determine the mechanism of protein expression in fetal liver sections of p53, Bcl-2, and Bax. Figure 3 shows a representative photograph showing p53, Bcl-2, and Bax expression (the original uncropped image is available in Supplementary Figure S1). The RG group had significantly higher levels of p53 and Bax expression (*p* < 0.05) than the CG, ARG, and NCG groups, and the ARG group had higher levels of these proteins than the CG and NC groups. However, the protein expression of p53 and Bax in the NCG group was not significantly different from that in the CG group. In contrast, Bcl-2 protein expression was significantly reduced (*p* < 0.05) in the RG group compared to the other groups, but not in the CG, ARG, or NCG groups.

## 4. Discussion

The restricted feeding treatment obviously affected fetal body and liver growth, and the supplementation with NCG partially ameliorated these indicators. The RP-Arg supplementation was able to maintain the fetal-liver to body-weight ratio. Under the condition of fetal undernutrition, the redistribution of the fetal cardiac output ensures brain development, but at the expense of the liver [23]. Moreover, the adaptation of the fetal liver is influenced by the severity of the mother’s nutritional deficit during pregnancy [3]. These changes are often referred to as fetal brain-sparing, a phenomenon which may also be responsible for the compromise of chemical component deposition in fetal liver [24]. Consistent with this view, the current study found that the basic chemical components of fetal livers were affected under the condition of nutritional restriction. Therefore, the growth and components of IUGR fetal liver were selectively affected under maternal undernutrition, but NCG supplementation in the maternal diet during pregnancy might ameliorate this asymmetric growth.

The DNA concentration in the fetal liver, as an index of hyperplasia, was altered by the maternal nutritional plane and NCG supplementation, and this was also the case for the liver RNA and protein concentrations. A previous study reported that the fetuses of nutrition-restricted ewes had lower protein concentrations in the liver, a finding that is in accordance with our results [18]. The increased ratio of protein to DNA may indicate that maternal NCG supplementation could increase cell size [10]; thus, we may conclude that fetal liver cellularity is a response to maternal NCG supplementation and nutritional levels. Further studies will be needed to investigate the reasons for the difference in the amount of fetal liver RNA and protein expression in more detail.

Fetal liver function has an important influence on fetal postnatal health. A previous study showed that IUGR fetuses had decreased blood levels of TP and ALT, but increased levels of AST, relative to controls [24]. These observations are in accordance with our results. Relative to nutrition-restricted ewes, we found that arginine supplementation, especially in the form of NCG, was able to improve the fetal serum concentrations of liver enzymes and TP. Thus, RP-Arg or NCG supplementation might improve liver function, which may also enhance hepatocellular integrity and increase protein synthesis [25]. Liver CHE concentrations may be influenced by nutritional status, and this indicator is also a marker of the overall functional reserve of the liver [26]. The reduction in serum CHE is reported to be significantly associated with liver dysfunction [27]. The decrease in the CHE concentration in the fetal livers from the restricted group may indicate that the liver function in fetuses is sensitive to maternal undernutrition, but the supplementation of maternal diet by RP-Arg or NCG could alleviate dysfunction.

The liver plays a key role in antioxidative defense, while oxidative stress affects the liver’s structure and function [28]. As a metabolic product of lipid peroxides, MDA has been found to be higher in the placental tissues of IUGR cases [29]. In the present study, the MDA concentration was significantly increased in the nutritionally restricted group, indicating a significant disruption in the oxidative balance of the fetal liver under maternal feed restriction. According to our findings, in the nutrition-restricted group, the GSH-Px concentration was increased to prevent more peroxide products, while the SOD activity was decreased in the fetal liver, indicating an increase in oxidative stress [30]. Moreover, the fetal liver tissues supplemented with RP-Arg or NCG had higher concentrations of SOD and TAC, indicating that RP-Arg or NCG could reduce oxidative stress and improve antioxidative capacity. A previous study showed that nitric oxide production is essential for hepatic lipid metabolism under starvation [31]. The low NO and NOS levels in the fetal liver from the restricted group only could indicate that supplementation with RP-Arg or NCG alleviated the feed-restriction-induced oxidative stress in the fetus through increasing NO and NOS levels in the liver. In this study, there was no difference in MAO levels in the livers among the groups; however, a slight decreasing trend was observed in the nutritionally restricted groups, which could indicate that maternal undernutrition may cause slight hepatic fibrosis in the fetal liver [32].

A previous study reported that apoptosis is necessary for the normal development of the fetus, and increased apoptosis is observed for as long as the pregnancy progresses [33]. However, apoptosis is also involved in a wide range of pathological processes, and its dysregulation is associated with numerous pathological indications [34]. Compared to the ARG and NCG groups, the control group had a significantly lower ratio of apoptotic TUNEL-positive cells, and the restricted group displayed the highest apoptosis rate. Consequently, our study indicates that maternal undernutrition during pregnancy causes abnormal liver development in the fetus due to increased apoptosis. We also measured the mRNA and protein expressions of some indicators that pivotally participate in the regulation mechanisms of apoptosis and anti-apoptosis. The mitochondrial pathway contains Bcl-2 family members (Bcl-2, Bcl-xL, and Bax) playing an integral role in the activation or repression of apoptosis in a variety of mammalian organs [35,36]. Moreover, cells die through apoptosis via the ratio of Bcl-2 to Bax, which is an important means of apoptosis regulation [37]. It has been proven that Bcl-2 can block the induction of apoptosis via p53 [36]. As such, p53 has been called a “cellular gatekeeper” or “the guardian of the genome”, and one of its most important functions is its ability to regulate apoptosis. The Fas receptor is a member of the tumor necrosis factor receptor superfamily, which induces apoptosis when oligomerized by its ligand (FasL) [38]. Several observations have pointed to Fas/FasL systems as key apoptotic signaling pathways [39]. Based on our results concerning the mRNA and protein expression of hepatocellular-apoptosis-regulating factors, we suggest that maternal undernutrition during pregnancy might cause increased apoptosis in the fetal liver, which affects the development of liver cells; moreover, the provision of RP-Arg or NCG supplementation to underfed pregnant ewes could improve the anti-apoptosis capacity of the fetal liver.

## 5. Conclusions

This study concludes that maternal undernutrition during pregnancy leads to the maldevelopment of the fetal liver. However, supplementation with RP-Arg and NCG can enhance fetal liver development under such conditions. This suggests the potential for nutritional interventions to improve fetal growth outcomes when maternal nutrition is compromised.

## Figures and Tables

**Table 1:** Basic characteristics of the fetal livers.

Items	Treatment Groups				SEM	*p*-Value
	CG	RG	ARG	NCG		
n.	16	16	16	16		
Fetal body weight, g	1877.88 ^a^	1400.75 ^c^	1659.25 ^b^	1681.75 ^b^	23.53	0.03
Liver, g	60.12 ^a^	51.18 ^c^	51.73 ^c^	55.34 ^b^	3.73	0.03
Body/liver weight (%)	3.19 ^b^	3.72 ^a^	3.14 ^b^	3.28 ^b^	0.41	0.02
Chemical components (g)						
Dry matter	12.56 ^a^	11.40 ^b^	11.44 ^b^	12.10 ^a^	0.31	0.03
Water	47.73 ^a^	40.69 ^c^	40.71 ^c^	43.13 ^b^	0.52	<0.01
Fat	1.17 ^b^	1.12 ^c^	1.19 ^bc^	1.27 ^a^	0.10	0.04
Protein	8.88 ^a^	8.29 ^b^	8.63 ^ab^	9.02 ^a^	0.38	0.03
Ash	0.61 ^a^	0.55 ^b^	0.60 ^a^	0.62 ^a^	0.07	0.50
Chemical components expressed as percentage of fetal liver weights (%)						
Dry matter	20.89 ^b^	22.27 ^a^	22.11 ^a^	21.86 ^a^	0.22	0.04
Water	79.39	79.50	78.70	77.94	0.32	0.49
Fat	1.95 ^b^	2.19 ^a^	2.30 ^a^	2.29 ^a^	0.05	<0.01
Protein	14.77 ^c^	16.20 ^b^	16.68 ^a^	16.30 ^b^	0.69	<0.01
Ash	1.01	1.07	1.16	1.12	0.05	0.73

Note: Without a common superscript letter (a–c), means differ (*p* < 0.05).

**Table 2:** Cellularity estimates for fetal livers.

Items	Treatment Groups				SEM	*p*-Value
	CG	RG	ARG	NCG		
DNA, mg/g	4.57 ^a^	4.32 ^c^	4.28 ^c^	4.50 ^b^	0.05	0.03
DNA, mg	0.26	0.21	0.22	0.24	0.01	0.57
RNA, mg/g	3.92 ^b^	3.63 ^c^	3.80 ^b^	4.10 ^a^	0.05	<0.01
RNA, mg	0.26 ^a^	0.20 ^b^	0.22 ^ab^	0.23 ^ab^	0.01	0.03
RNA/DNA	1.06	1.04	1.10	1.13	0.02	0.30
Protein, mg/g	54.63 ^a^	48.53 ^c^	51.53 ^b^	55.83 ^a^	0.88	<0.01
Protein, mg	3.28 ^a^	2.53 ^b^	2.72 ^b^	3.15 ^a^	0.10	<0.01
Protein/DNA	11.17 ^a^	10.34 ^b^	10.58 ^b^	10.92 ^ab^	0.11	0.02

Note: Without a common superscript letter (a–c), means differ (*p* < 0.05).

**Table 3:** Liver function indicators and antioxidant activities of fetal serum and liver extract samples.

Items	Treatment Groups				SEM	*p*-Value
	CG	RG	ARG	NCG		
Fetal serum						
ALT (IU/L)	5.36 ^a^	3.28 ^c^	4.11 ^b^	4.78 ^a^	0.25	<0.01
AST (IU/L)	25.56 ^c^	37.11 ^a^	31.83 ^b^	26.86 ^c^	1.38	<0.01
TP (g/L)	41.48 ^a^	31.03 ^c^	33.91 ^bc^	36.04 ^b^	1.17	<0.01
Fetal liver extract						
CHE (U/mg)	11.81 ^a^	8.57 ^b^	11.96 ^a^	11.41 ^a^	0.43	<0.01
GSH-Px (U/mg)	26.88 ^b^	34.01 ^a^	27.23 ^b^	25.56 ^b^	1.03	<0.01
MAO (U/mg)	2.13	2.10	2.11	2.10	0.02	0.94
MDA (nmol/mg)	4.07 ^b^	5.18 ^a^	4.43 ^b^	4.06 ^b^	0.14	0.04
NO (umol/g)	2.29 ^a^	1.69 ^b^	2.38 ^a^	2.68 ^a^	0.11	<0.01
NOS (U/mg)	0.84 ^a^	0.65 ^c^	0.70 ^bc^	0.78 ^ab^	0.03	0.03
SOD (U/mg)	26.39 ^a^	21.61 ^b^	22.87 ^b^	24.92 ^ab^	0.59	0.01
TAC (U/mg)	2.65 ^a^	1.67 ^c^	2.28 ^b^	2.59 ^a^	0.12	<0.01

Note: ALT = alanine aminotransferase; AST = aspartate aminotransferase; TP = total protein; CHE = cholinesterase; GSH-Px = glutathione peroxidase; MAO = monoamine oxidase; MDA = maleic dialdehyde; NO = nitric oxide; NOS = nitric oxide synthase; SOD = superoxide dismutase; TAC = total antioxidant capacity. Without a common superscript letter (a–c), means differ (*p* < 0.05).

## Data Availability

Data are contained within the article.

## Supplementary Materials

The following supporting information can be downloaded at: https://www.mdpi.com/article/10.3390/ani14131988/s1, Figure S1: Original Western blot figure. Table S1: Ingredient and nutrient composition of the experimental diets on a DM basis.; Table S2. Primer sequences used in qRT-PCR.
