# Supplementary material for: Dietary Supplementation with Rumen-Protected Arginine or N-Carbamylglutamate Enhances Fetal Liver Development in Nutrient-Restricted Pregnant Hu Ewes
Source: Animals (Basel). 2024 Jul 5;14(13):1988. doi: 10.3390/ani14131988 (PMC11240601; doi:10.3390/ani14131988)

Supplemental Table

Table S1. Ingredient and nutrient composition of the experimental diets on a DM basis.

Item	Diet 1	Diet 2
	0 to 90 d of gestation	91 to 110 d of gestation
Ingredient, %		
Chinese wild rye	50.0	45.0
Corn	35.12	31.32
Soybean meal	12.00	20.00
Dicalcium phosphate	1.67	2.34
Calcium carbonate	0.41	0.54
Salt	0.50	0.50
Mineral/vitamin premix[1]	0.30	0.30
Total	100	100
Nutrient composition (analyzed)		
GE, MJ/kg	17.63	18.49
CP, %	9.98	13.59
Ether extract, %	4.21	4.59
NDF, %	37.12	32.57
ADF, %	20.98	18.93
Ca, %	0.57	0.81
P, %	0.45	0.69

[1]The premix provided the following nutrients per kg of the diet: 30,000 IU vitamin A, 10,000 IU vitamin D, 100 mg vitamin E, 90 mg Fe, 12.5 mg Cu, 50 mg Mn, 100 mg Zn, 0.3 mg Se, 0.8 mg I, and 0.5 mg Co.

Table S2. Primer sequences used in qRT-PCR.

Gene	GenBank Accession numer	Primer sequences (5′-3′)		Annealing temperature (°C)	Length (bp)
		Forward primer sequence	Reverse primer sequence		
GAPDH	NM_001034034.1	GTCAAGGCAGAGAACGGGAA	GGTTCACGCCCATCACAAAC	59	119
p53	X81705.1	CCCGCCTCAGCACCTTAT	GCACAAACACGCACCTCA	58	261
Fas	NM_001123003.1	TTTTGCTGTCAGCCTTGTCC	TGTTCCACTTCTAGCCCATG	57	179
FasL	XM_004013705.1	TCTGTGGAGAAGCAAATAGGTC	AGGGCAATTCCATAGGTGTC	57	134
Bcl-2	DQ152929.1	CGCATCGTGGCCTTCTTT	CGGTTCAGGTACTCGGTCATC	59	62
Bax	AF163774.1	CGAGTGGCGGCTGAAAT	GGTCTGCCATGTGGGTGTC	59	238

Supplemental Figure S1. Original Western Blot figure

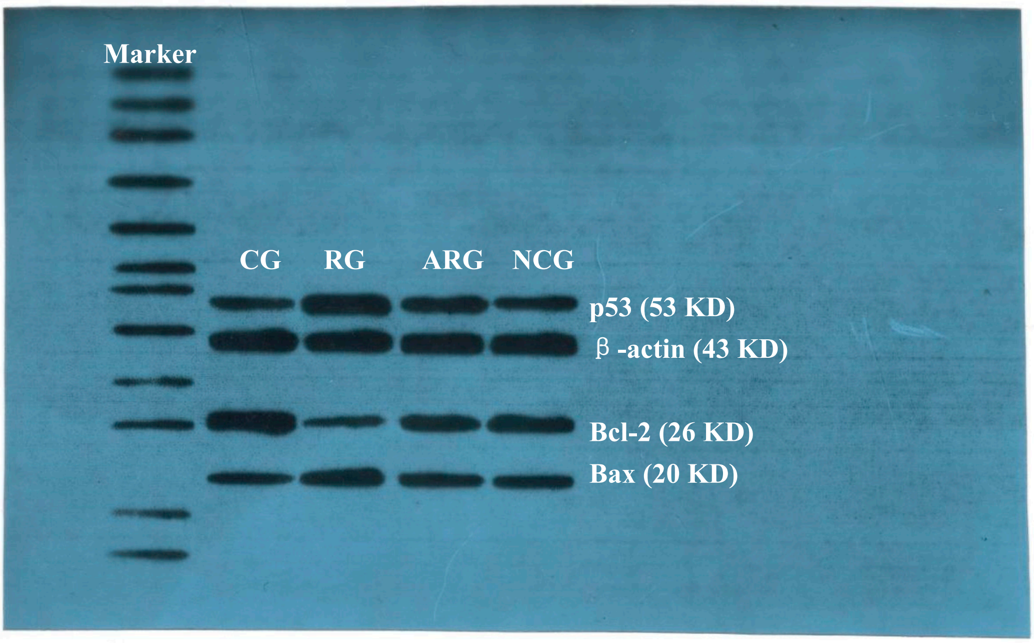

Supplement: Supplementary file 1 [file animals-14-01988-s001.zip › animals-3053790-supplementary.pdf]
